# Systemic and Splanchnic Lipopolysaccharide and Endothelin-1 Plasma Levels in Liver Cirrhosis before and after Transjugular Intrahepatic Portosystemic Shunt

**DOI:** 10.1155/2016/8341030

**Published:** 2016-01-31

**Authors:** Jiaxiang Meng, Qing Wang, Kai Liu, Shuofei Yang, Xinxin Fan, Baochen Liu, Changsheng He, Xingjiang Wu

**Affiliations:** ^1^Department of General Surgery, Jinling Hospital, Medical School of Nanjing University, Nanjing, Jiangsu 210002, China; ^2^State Key Laboratory of Oncogenes and Related Genes, Shanghai Cancer Institute, Renji Hospital, School of Medicine, Shanghai Jiao Tong University, Shanghai 200030, China; ^3^Research Institute of General Surgery, Jinling Hospital, Medical School of Nanjing University, Nanjing, Jiangsu 210002, China

## Abstract

Lipopolysaccharide (LPS) and endothelin- (ET-) 1 may aggravate portal hypertension by increasing intrahepatic resistance and splanchnic blood flow. In the portal vein, after TIPS shunting, LPS and ET-1 were significantly decreased. Our study suggests that TIPS can benefit cirrhotic patients not only in high hemodynamics related variceal bleeding but also in intestinal bacterial translocation associated complications such as endotoxemia.

## 1. Introduction 

Portal hypertension is a complication of liver cirrhosis. Cirrhotic nodules lead to altered intrahepatic architecture and are the initiating, irreversible pathophysiological feature of cirrhosis. The major pathophysiological mechanisms of portal hypertension are increased intrahepatic resistance and increased splanchnic blood flow, so intrahepatic vascular contraction and increased splanchnic blood flow may be key therapeutic targets in portal hypertension [1].

Bacterial translocation is a common problem and plays an important role in the pathogenesis and complications in patients with decompensated cirrhosis [2]. Bacterial endotoxin, such as lipopolysaccharide (LPS), is a prototypic microbe-derived inflammatory signal that regulates endothelin (ET) and NO synthesis [3]. The regulation is complex in advanced cirrhosis. Circulating LPS may have an important role in inducing intrahepatic sinusoidal and splanchnic vascular endothelial dysfunction. ET-1 is one of the most potent vasoconstrictors and binds to ET-A and ET-B receptors. ET-A receptors are typically located on vascular smooth muscle cells and mediate vasoconstriction, whereas ET-B receptors on endothelial cells stimulate endothelial NO synthase (eNOS) activity and NO release. ET-1 may play an important role in liver disease, especially in circulatory disorders such as portal hypertension and ischemia [4].

Transjugular intrahepatic portosystemic shunt (TIPS) is an important interventional procedure for treatment of the complications of advanced cirrhosis that have failed with medical management [5]. TIPS reduces the portal venous pressure gradient (PVPG) and gives the opportunity to obtain portal and hepatic venous blood directly, to determine concentrations of LPS and ET-1 and evaluate their contribution to intrahepatic and systemic hemodynamics. Thus, the aim of this study was to determine portal, hepatic, and systemic LPS and plasma ET-1 levels before and after TIPS in cirrhotic patients to better understand the portal hypertension and its complications.

## 2. Patients and Methods

### 2.1. Patients

We studied 30 consecutive patients with portal hypertension at high risk of acute variceal bleeding who underwent TIPS at Jinling Hospital, Nanjing, China, between October 2013 and December 2014. Patients had severe esophageal varices upon endoscopy, had more than one episode of variceal bleeding, and had failed drug or endoscopic treatment. Diagnosis of cirrhosis was established by a combination of biochemical, clinical, ultrasonographic, and liver histological findings. The etiology of cirrhosis was alcohol in three patients, chronic Budd-Chiari syndrome in three patients, and viral hepatitis B in 24 patients. No alcohol abuse was detected 2 months before the procedure. The clinical indications for TIPS include repeated variceal bleeding despite appropriate secondary prophylaxis (*n* = 25) and ascites refractory to conventional treatment (*n* = 5).

### 2.2. Study Design

**Figure 1:**
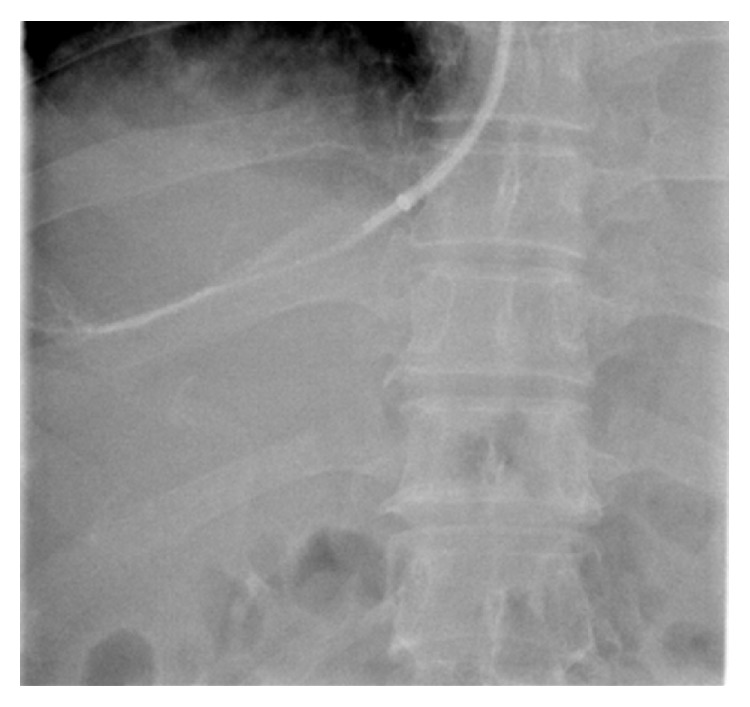
Hepatic venous blood samples were taken under guidance of X-rays.

**Figure 2:**
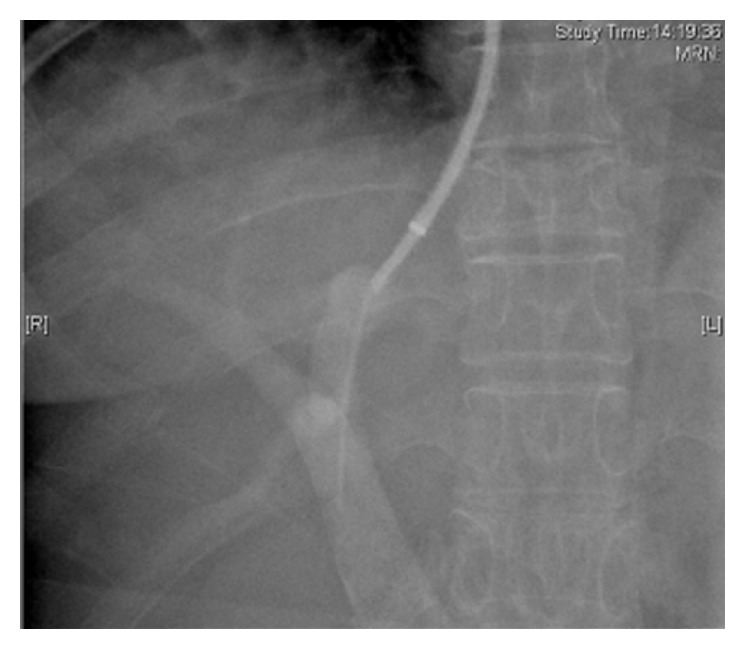
Portal venous blood samples were taken under guidance of X-rays.

Blood samples were taken from the right atrium, hepatic vein, and portal vein before insertion of the TIPS stent and 7 days after the TIPS procedure (Figures 1 and 2). Plasma samples were centrifuged at 1800 g for 15 min at 4°C and immediately stored at −80°C until they were analyzed. Serum LPS and ET-1 were measured by enzyme linked immunosorbent assay (ELISA) as previously described [6]. Control samples and serum standards were jointly analyzed in each run. The interassay coefficient of variation in the current study (six runs) was ~10%. Serum NO was measured from the nitrate/nitrite content using a fluorometric assay (KGE 001; R&D Systems China, Shanghai, China). All other analyses were performed using standard laboratory methods.

### 2.3. TIPS

TIPS was performed as described previously [7]. Stents were grafts covered with extended polytetrafluoroethylene (Fluency; BARD Peripheral Vascular, Tempe, AZ, USA) and inserted according to general guidelines. The covered stents were 8 or 10 mm in diameter. PVPG was measured during the procedure and 7 days after TIPS. The measurement of PVPG and the acquisition of different blood samples were conducted under the guidance of X-rays (Figures 1 and 2). Intravenous administration of a prophylactic broad-spectrum antibiotic was used after taking blood samples.

### 2.4. Statistical Analysis

Statistical analysis was performed with SPSS for Windows version 17. Quantitative variables are displayed as medians if not otherwise indicated. We used Student's *t*-test for comparing differences among continuous normally distributed data and a *χ* ^2^ test for categorical data. For analysis of correlation, we calculated the Spearman coefficient of correlation. Differences with *P* < 0.05 were considered significant.

### 2.5. Ethical Considerations

The Ethics Committee of Jinling Hospital approved this study. Written informed consent was obtained from each patient prior to the study.

## 3. Results

### 3.1. Patient Characteristics

The demographic and biochemical characteristics of the patients are listed in Table 1. TIPS was successfully placed in all of the patients. PVPG was significantly lowered from a median of 18 (range 12–32) to 10 (8–16) mmHg (*P* < 0.05). Three patients had transient, low-grade hepatic encephalopathy that was manageable by diet and laxatives without shunt reduction. No patient experienced upper gastrointestinal bleeding after TIPS during 6–13 (median 8) months of follow-up.

### 3.2. LPS and ET-1 in Portal and Hepatic Veins

**Figure 3:**
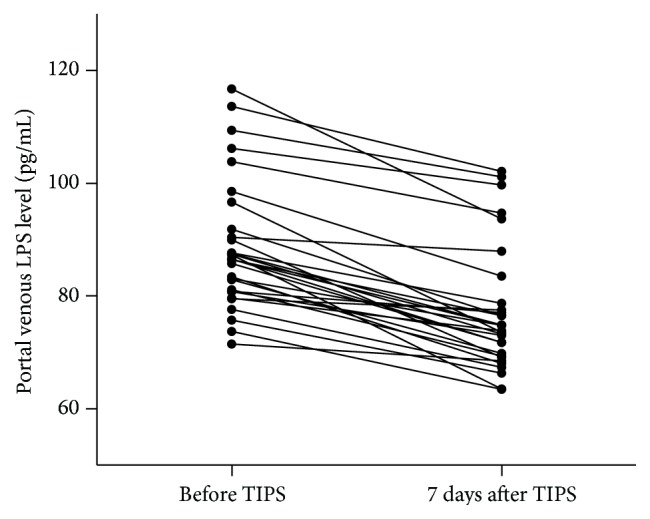
Portal venous plasma LPS concentration before and 5–7 days after TIPS procedure depicted as dot plots, illustrating median, range, and 50% interval with 25th and 75th percentile. The level of LPS in portal vein was decreased from 88 ± 8.63 to 77 ± 7.32 pg/mL (*P* < 0.05).

**Figure 4:**
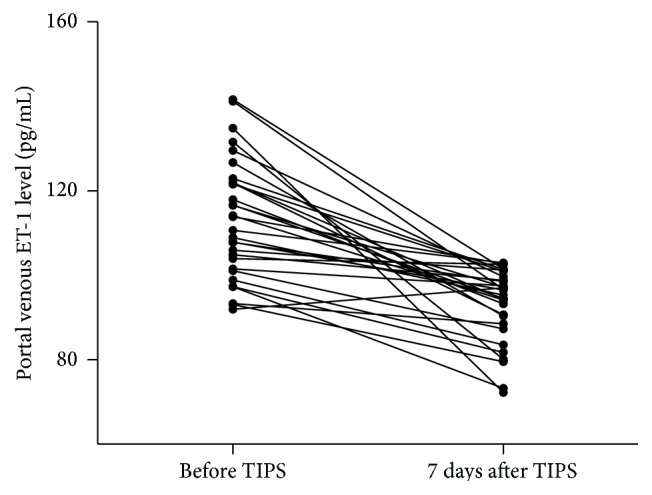
Portal venous plasma ET-1 concentration before and 5–7 days after TIPS procedure depicted as dot plots, illustrating median, range, and 50% interval with 25th and 75th percentile. The level of ET-1 in portal vein was decreased from 113 ± 3.51 to 93 ± 9.31 pg/mL (*P* < 0.05).

Before TIPS, LPS level did not differ significantly between portal vein and hepatic vein plasma: 88 (56–105) versus 92 (54–110) pg/mL. In the portal vein, LPS level decreased significantly from 88 ± 8.63 to 77 ± 7.32 pg/mL (*P* < 0.05) (Figure 3) after TIPS placement. The level of ET-1 also decreased significantly from 113 ± 3.51 to 93 ± 9.31 pg/mL (*P* < 0.05) (Figure 4). There was no difference in the concentration of NO in the portal vein after TIPS placement (from 32.5 to 34.3 pg/mL; *P* = 0.076). Subgroup analysis demonstrated that median portal venous plasma LPS and ET-1 levels before TIPS were significantly higher in five patients with refractory ascites [113 (98–132) pg/mL] compared with 25 patients with repetitive variceal bleeding [86 (56–98) pg/mL] (*P* < 0.05).

### 3.3. LPS and PVPG after TIPS

**Figure 5:**
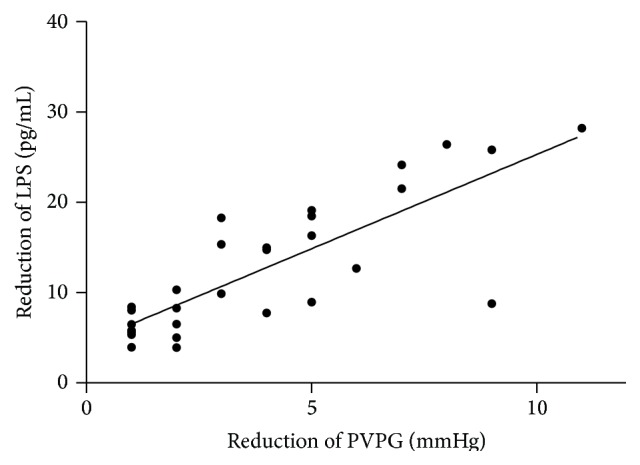
The reduction level of LPS was correlated with the reduction of the PVPG 7 days after the TIPS insertion (Spearman's *r* = 0.67; *P* < 0.05).

Regression analysis showed no significant correlation between right atrial and portal venous levels of LPS and ET-1 and PVPG before and after TIPS insertion. However, when compared with the PVPG at the time during TIPS insertion and 7 days after TIPS, PVPG decreased significantly from 10 (8–16) to 8 (6–14) mmHg (*P* < 0.05). From the time before TIPS and after TIPS insertion, there was a significant correlation between the reduction in portal venous LPS and the reduction in PVPG (Spearman's *r* = 0.67; *P* < 0.05) (Figure 5).

## 4. Discussion

The main findings of the present study were as follows. We found no intrahepatic gradient of LPS before TIPS placement. LPS and ET-1 levels were decreased in the portal vein after TIPS insertion. During the time before TIPS and after TIPS, there was a correlation between the reduction in portal venous LPS and the reduction in PVPG.

Bacterial translocation is defined as the passage of both viable and nonviable bacteria and bacterial products, such as endotoxin. It is common in decompensated cirrhosis and may be an important pathogenic event in several complications of cirrhosis [1]. LPS is a surrogate marker of bacterial translocation and is increased in systemic and portal circulation [8]. In this study, we did not find a significant difference in LPS levels between the portal and hepatic veins, which is in consistence with Trebicka et al. study [9]. The lack of hepatic endotoxin gradient in our patients may have resulted from the presence of extrahepatic collateral vessels and impaired liver function. We did not find a significant difference in right atrial blood LPS levels before and after stent insertion. However, a recent study showed that TIPS increased LPS levels in peripheral blood 1 h after stent placement in patients with acute, uncontrolled bleeding [10]. This phenomenon might result from short-term hemodynamic changes caused by procedural trauma or acute bleeding [11].

We found reduced LPS levels in the portal vein; thus, TIPS may reduce LPS levels in the portal vein after stent insertion, possibly as a result of reducing the PVPG. Portal hypertension may be an important factor in the development of small bowel mucosal changes [12]. Abraldes et al. demonstrated that portal pressure is sensed in different vascular beds depending on the severity of portal hypertension, and small increases in portal pressure are first sensed by the intestinal microcirculation [13]. In patients with cirrhosis and portal hypertension, small bowel mucosal edema, red spots, and small bowel varices are attenuated after TIPS [14].

We found that ET-1 level was decreased in the portal vein after TIPS insertion. ET-1 may play an important role in liver disease, especially in circulatory disorders such as portal hypertension and ischemia. ET-1 acts as a paracrine hormone and its plasma levels could represent an overflow of locally produced peptides [15]. Fluid shear stress is a strong liberator of ET-1 from splanchnic vascular endothelial cells, and TIPS could markedly reduce the PVPG and fluid shear stress [16]. Kawanaka et al. found that splenectomy reduced portal venous pressure and normalized hepatic concentrations of ET-1 in patients with liver cirrhosis and portal hypertension. Splenectomy may decrease systemic and splanchnic circulation by eliminating spleen-derived ET-1. Vascular endothelial cells in enlarged spleen may be an important source of ET-1, and TIPS could reduce portal pressure and improve the enlarged spleen [17].

The reduction in portal venous LPS was well correlated with the reduction in PVPG after the TIPS procedure. Binding of ET-1 to ET-B receptors results in activation of eNOS and production of NO, which lead to vasodilation at the sinusoidal level [1]. During endotoxemia, the liver microcirculation becomes hypersensitive to ET-1-induced vasoconstriction. LPS inhibits ET-1-induced eNOS activation in hepatic sinusoidal cells. Therefore, the decrease in ET-1 and LPS levels in the portal vein may reduce intrahepatic vascular resistance owing to NO production in endothelial cells mediated by ET-B receptors [18].

It should be noted that the concentrations in our study have a preliminary character because of the limited numbers and the heterogeneous nature of the patients (e.g., different Child-Pugh classes, ascites, and acute bleeding). Additionally, the blood samples were measured before and 7 days after TIPS insertion separately, so the concentration may be attributed to the different times when the blood samples were obtained [9, 10].

In conclusion, we observed that, after the TIPS procedure, LPS and ET-1 levels in the portal vein both were decreased and the reduction in portal venous LPS was well correlated with the reduction in PVPG. Our study suggests that TIPS can benefit cirrhotic patients not only in high hemodynamics related variceal bleeding but also in intestinal bacterial translocation associated complications such as endotoxemia.

## Figures and Tables

**Table 1:** Basic characteristics of patients (plus/minus are means ± SD).

Gender (male/female)	24/6
Age (years)	52.5 ± 11.8
INR	1.3 (1.1–1.48)
Serum ALAT (U/L)	26.8 ± 13.5
Plasma albumin (g/L)	36 ± 4.29
Plasma creatinine (s)	87 (50–108)
Child-Pugh class (A/B/C)	5/18/7
Cirrhosis aetiology (*n*)	
HBV	24
Chronic Budd-Chiari	3
Alcohol	3
Indication	
Refractory ascites	25
Recurrent variceal bleeding	19

AST: aspartate aminotransferase; INR: international normalized ratio.

## List of Abbreviations

### Abbreviations

LPS: Lipopolysaccharide
ET-1: Endothelin-1
TIPS: Transjugular intrahepatic portosystemic shunt
ELISA: Enzyme linked immunosorbent assay
NO: Nitric oxide
eNOS: Endothelial nitric oxide synthase
PVPG: Portal venous pressure gradient.
